# Supplementary material for: Instrumental music training relates to intensity assessment but not emotional prosody recognition in Mandarin
Source: PLoS One. 2024 Aug 30;19(8):e0309432. doi: 10.1371/journal.pone.0309432 (PMC11364251; doi:10.1371/journal.pone.0309432)
Supplement: S2 Table — (DOCX) [file pone.0309432.s002.docx]

# S2 Table. Pairwise comparisons of emotional prosodies.

| **Comparison** | ***t*(20)** | ***d*** | **95% CI** | ***BF*** |
| --- | --- | --- | --- | --- |
| **Recognition rates** |  |  |  |  |
| Happiness vs. Sadness | −4.28 | −0.96 | [−1.48, −0.42] | 87.45 |
| Happiness vs. Fear | −1.54 | −0.34 | [−0.79, 0.11] | 0.63 |
| Happiness vs. Anger | −2.97 | −0.66 | [−1.14, −0.17] | 6.37 |
| Happiness vs. Neutrality | −4.57 | −1.02 | [−1.55, −0.47] | 159.13 |
| Sadness vs. Fear | 1.75 | 0.39 | [−0.07, 0.84] | 0.83 |
| Sadness vs. Anger | 0.98 | 0.22 | [−0.23, 0.66] | 0.35 |
| Sadness vs. Neutrality | −0.36 | −0.08 | [−0.52, 0.36] | 0.24 |
| Fear vs. Anger | −1.23 | −0.28 | [−0.72, 0.17] | 0.44 |
| Fear vs. Neutrality | −2.48 | −0.55 | [−1.02, −0.08] | 2.62 |
| Anger vs. Neutrality | −0.75 | −0.17 | [−0.61, 0.28] | 0.29 |
| **Intensity ratings** |  |  |  |  |
| Happiness vs. Sadness | −4.99 | −1.12 | [−1.66, −0.55] | 378.56 |
| Happiness vs. Fear | −2.99 | −0.67 | [−1.15, −0.18] | 6.62 |
| Happiness vs. Anger | −4.66 | −1.04 | [−1.58, −0.49] | 191.65 |
| Sadness vs. Fear | 3.36 | 0.75 | [0.25, 1.24] | 13.54 |
| Sadness vs. Anger | 2.30 | 0.51 | [0.04, 0.97] | 1.93 |
| Fear vs. Anger | −1.14 | −0.25 | [−0.70, 0.19] | 0.40 |
