# Supplementary material for: Instrumental music training relates to intensity assessment but not emotional prosody recognition in Mandarin
Source: PLoS One. 2024 Aug 30;19(8):e0309432. doi: 10.1371/journal.pone.0309432 (PMC11364251; doi:10.1371/journal.pone.0309432)
Supplement: S3 Table — All statistics were obtained from the aligned-and-ranked data. (DOCX) [file pone.0309432.s003.docx]

# S3 Table. Pairwise comparisons of emotional prosodies.

| **Comparison** | ***t*(280)** | ***d*** | **95% CI** | ***BF*** |
| --- | --- | --- | --- | --- |
| **BIS** |  |  |  |  |
| Happiness vs. Sadness | −0.76 | −0.05 | [−0.16, 0.07] | 0.23 |
| Happiness vs. Fear | 6.38 | 0.38 | [0.26, 0.50] | 6.42 × 10^4^ |
| Happiness vs. Anger | 1.06 | 0.06 | [−0.05, 0.18] | 0.30 |
| Happiness vs. Neutrality | −5.22 | −0.31 | [−0.43, −0.19] | 2.45 × 10^3^ |
| Sadness vs. Fear | 7.14 | 0.43 | [0.30, 0.55] | 5.41 × 10^5^ |
| Sadness vs. Anger | 1.82 | 0.11 | [−0.01, 0.23] | 0.79 |
| Sadness vs. Neutrality | −4.46 | −0.27 | [−0.39, −0.15] | 302.09 |
| Fear vs. Anger | −5.33 | −0.32 | [−0.44, −0.20] | 3.33 × 10^3^ |
| Fear vs. Neutrality | −11.60 | −0.69 | [−0.82, −0.56] | 4.97 × 10^10^ |
| Anger vs. Neutrality | −6.27 | −0.37 | [−0.50, −0.25] | 4.71 × 10^4^ |
| **Intensity ratings** |  |  |  |  |
| Happiness vs. Sadness | 0.49 | 0.03 | [−0.09, 0.15] | 0.20 |
| Happiness vs. Fear | 2.60 | 0.16 | [0.04, 0.27] | 3.27 |
| Happiness vs. Anger | −5.56 | −0.33 | [−0.45, −0.21] | 6.36 × 10^3^ |
| Happiness vs. Neutrality | 11.25 | 0.67 | [0.54, 0.80] | 2.20 × 10^10^ |
| Sadness vs. Fear | 2.11 | 0.13 | [0.01, 0.24] | 1.28 |
| Sadness vs. Anger | −6.05 | −0.36 | [−0.48, −0.24] | 2.53 × 10^4^ |
| Sadness vs. Neutrality | 10.77 | 0.64 | [0.51, 0.77] | 7.02 × 10^9^ |
| Fear vs. Anger | −8.16 | −0.49 | [−0.61, −0.36] | 8.89 × 10^6^ |
| Fear vs. Neutrality | 8.65 | 0.52 | [0.39, 0.64] | 3.29 × 10^7^ |
| Anger vs. Neutrality | 16.81 | 1.00 | [0.86, 1.15] | 2.02 × 10^15^ |

All statistics were obtained from the aligned-and-ranked data.
