# Supplementary material for: Instrumental music training relates to intensity assessment but not emotional prosody recognition in Mandarin
Source: PLoS One. 2024 Aug 30;19(8):e0309432. doi: 10.1371/journal.pone.0309432 (PMC11364251; doi:10.1371/journal.pone.0309432)
Supplement: Attachment — Submitted filename: Response to Reviewers.docx [file pone.0309432.s004.docx]

# Response to Academic Editor and Reviewers

## Academic Editor

Journal requirements:

When submitting your revision, we need you to address these additional requirements.

1. Please ensure that your manuscript meets PLOS ONE's style requirements, including those for file naming. The PLOS ONE style templates can be found at https://journals.plos.org/plosone/s/file?id=wjVg/PLOSOne_formatting_sample_main_body.pdf and https://journals.plos.org/plosone/s/file?id=ba62/PLOSOne_formatting_sample_title_authors_affiliations.pdf

**Response:** We have made the changes required by PLOS ONE's style.

2. When completing the data availability statement of the submission form, you indicated that you will make your data available on acceptance. We strongly recommend all authors decide on a data sharing plan before acceptance, as the process can be lengthy and hold up publication timelines. Please note that, though access restrictions are acceptable now, your entire data will need to be made freely accessible if your manuscript is accepted for publication. This policy applies to all data except where public deposition would breach compliance with the protocol approved by your research ethics board. If you are unable to adhere to our open data policy, please kindly revise your statement to explain your reasoning and we will seek the editor's input on an exemption. Please be assured that, once you have provided your new statement, the assessment of your exemption will not hold up the peer review process.

**Response:** The data, stimuli, and procedure of this study are publicly available for download from data storage repositories.

3. Please include captions for your Supporting Information files at the end of your manuscript, and update any intext citations to match accordingly. Please see our Supporting Information guidelines for more information: http://journals.plos.org/plosone/s/supporting-information.

**Response:** Done.

While revising your submission, please upload your figure files to the Preflight Analysis and Conversion Engine (PACE) digital diagnostic tool, https://pacev2.apexcovantage.com/. PACE helps ensure that figures meet PLOS requirements. To use PACE, you must first register as a user. Registration is free. Then, login and navigate to the UPLOAD tab, where you will find detailed instructions on how to use the tool. If you encounter any issues or have any questions when using PACE, please email PLOS at figures@plos.org. Please note that Supporting Information files do not need this step.

**Response:** Done.

## Reviewer #1

There is a lot to like about this study: the research question is well motivated, the sample size is appropriate, the two groups of participants are carefully matched on demographic and cognitive variables, the methods are sound, and the analyses are carefully conducted based on both frequentist and Bayesian statistics. I also think that it is important to publish these null results, particularly considering previous mixed findings obtained with speakers of nontonal languages.

My main concern is with the causal framework adopted by the authors, seen throughout the manuscript from the title (‘Impact of instrumental music training’) to the last paragraph of the discussion. Although they acknowledge briefly that their design precludes inferences of causation, their entire reasoning suggests otherwise. I think that the focus should be on what the study is about – an association – and that the issue of causation needs to be discussed in more detail and critically. Recent reviews of the literature, both focused on emotion recognition specifically (Martins et al., 2021, Emotion Review; Nussbaum & Schweinberger, 2021, Emotion Review), and on music training effects more broadly (Schellenberg & Lima, 2024, Annual Review of Psychology), are directly relevant to this issue.

**Response:** We acknowledge your concerns regarding the causal framework initially suggested in our work. In light of your feedback and the cited literature, we have made careful revisions to more accurately reflect the associative nature of our findings across the manuscript. Furthermore, we have expanded our discussion section to critically address the limitations of our experimental design and its capacity for causal inference, enhancing our manuscript’s rigor.

Other points:

- In the Introduction, the assertion that emotional prosody recognition is important for social interactions would benefit from direct supporting evidence, instead of the indirect evidence that the authors cite (e.g., Neves et al., 2021, Royal Society Open Science).

**Response:** We have replaced the indirect evidence with the direct evidence (e.g., Chronaki et al., 2018; Neves et al., 2021) as suggested. (p. 3, ll. 34-35)

- On p. 4, the sentence starting on line 71 lacks an ending.

**Response:** Thanks for pointing out this. We have modified this sentence to read “Many studies have examined the relationship between music training and emotional prosody recognition in native speakers of nontonal languages, such as English, German, Portuguese, Dutch, and Hebrew.”

- On p. 5, line 81, the authors say that ‘experimental studies generally support the enhancement of emotional prosody recognition through mixed music training’. The studies that they cite do not provide evidence for such effect, though. This needs to be clarified.

**Response:** Thanks for your astute observation. For the sake of contextual fluency, we have deleted this sentence and the two references you mention in this revision. (p. 5, l. 82)

- Why were the stimuli produced by male and female speakers presented in separate blocks?

**Response:** We presented male and female speaker stimuli in separate blocks for two main reasons. First, to control for potential gender-related differences in vocal characteristics. Male and female voices naturally differ in acoustic properties such as pitch and timbre (Hillenbrand & Clark, 2009; Santos et al., 2023). By separating the blocks, we ensure that any observed differences in participants’ responses to emotional prosody are attributed to the emotional content itself rather than being confounded by the speaker’s gender. Second, to reduce participants’ cognitive load. Presenting a large number of stimuli with varying emotions and speaker genders can be cognitively demanding. Separating the stimuli helps ensure that participants are not distracted or influenced by sudden changes in speaker gender, allowing for better focus on individual emotions, reducing fatigue, and improving overall task performance. This has already been clarified in the text. (p. 14, ll. 230-232)

- Although I understand the rationale for using a composite metric that incorporates speed and accuracy, for comparability with previous studies it would be useful to also present analyses (and descriptives) just focused on accuracy.

**Response:** Following your suggestion, we have included the results for both accuracy and response time in the revised manuscript, aligning with previous research and enhancing the comparability of our findings. (p. 15, ll. 257-258; pp. 19-20, ll. 328-351)

- Considering that the novel aspect of this study is the focus on a tonal language, I missed a detailed discussion of how/why differences in linguistic experience could moderate music training effects in prosody recognition. The authors allude to this issue only superficially, without specifying potential reasons for a pattern of null results in tonal languages and predominantly positive results in nontonal ones.

**Response:** Thanks for the reminder. We have addressed how linguistic experience could moderate the effects of music training on emotional prosody recognition. Studies show that 53%-72% of Chinese music subjects speaking a tonal language had absolute pitch ability, which may not benefit emotional prosody recognition that relies more on relative pitch processing. Conversely, the majority of musicians speaking nontonal languages exhibit stronger relative pitch skills compared to non-musicians. This enhanced relative pitch ability likely contributes to their superior emotional prosody recognition. (p. 24, ll. 437-448)

## Reviewer #2

This study is interesting. Despite many previous studies on the relationship between language and music, much debate as to what role such relationships play in daily life. This study approached this unclear issue from the perspectives of the characteristics of language. On the other hand, several points may be difficult for potential readers to understand, so please consider the following points.

p.5, l.96-101 “Recognizing that lexical tones are a common element in both language and music [54] and acknowledging the disparities in acoustic cues employed by listeners of nontonal and tonal languages when interpreting lexical tones [55, 56], it remains unclear whether music training aids in the perception of emotional prosody among native speakers of tonal languages. To fill this gap in current research, the present study aims to explore the connection between music training and the recognition of emotional prosody in tonal languages.” Comment 1: Given that language differences are one of the main appeal points of this study, the aim of the current study can include international comparisons. If cultural contrasts are included in the scope of the study, the research would be more extensive.

**Response:** Thank you for your insightful suggestions regarding the inclusion of international comparisons and the exploration of cultural contrasts. Your comments have helped us recognize the importance of broadening the scope of our research to incorporate a more nuanced analysis of how cultural backgrounds influence the processing and interpretation of emotional cues in different language systems.

In response to your recommendations, we have revised the relevant sections of our manuscript to highlight the disparities in acoustic cue utilization between listeners of tonal and nontonal languages and to discuss the varying reliance on verbal versus semantic cues across different cultures. This revision not only enriches our discussion of the existing literature but also sets a more comprehensive context for our investigation into the impact of musical training on the perception of emotional prosody. (pp. 5-6, ll. 97-101)

p.17, l.362-371 Comment 2: To shed light on differences of the paper from other studies, the first paragraph of the Discussion should provide a sufficient summary of the appeal points of this study, such as ‘statistical methods’ and ‘large sample size’. In addition, the appeal points summarized here should also be reflected in the abstract.

**Response:** Thanks for your suggestions. As recommended, we have incorporated the unique contributions regarding sample size and methodology of our study in both the discussion section and the abstract. (p. 2, ll. 20-25; p. 23, ll. 417-419)

p.18, l.366 “Interestingly, music majors assigned higher intensity ratings to happy, fearful, and angry prosodies.” Comment 3: Why were differences observed in these three intensities? It would be important to elucidate this cause within Discussion for explanations of the overall results.

**Response:** Thank you for your reminder. In this revised version, we have addressed the reasons why music majors assigned higher intensity ratings to happy, fearful, and angry prosodies. We propose two possible explanations:

1. **Enhanced Auditory Perception:** Music majors' heightened auditory abilities may lead them to pick up on subtle cues in the music that influence the perceived intensity of emotion.
2. **Attentional Abilities:** Their training may enhance their attention to emotional cues, making them more attuned to the emotional content of the prosodies, particularly high-arousal emotions like happiness, fear, and anger.

This dual effect may lead to music majors experiencing these high-arousal emotions as more intense than those with low arousal ones. (pp. 25-26, ll. 459-475)

p.18, l.380-382 “This inconsistency may be explained by the intricate interplay between music and language experience in shaping emotional prosody recognition” Comment 4: In several studies of absolute pitch, the high number of absolute pitch holders in the Asian region is sometimes explained by linguistic characteristics. These findings of absolute pitch may also provide clues for interpreting the present results.

**Response:** We have discussed the possible impact of absolute pitch on our results. (p. 24, ll. 437-448)

p.19, l.393-394 “The discrepancy may be attributed to the difference in sample sizes;” Comment 5: Are these differences really only due to sample size? The differences due to language can be possible. If so, why do they differ by language? These factors should be discussed more carefully. This would deepen crosscultural aspects in this research topic.

**Response:** Thank you for kindly reminding us to consider this possibility. However, the observed contradiction likely doesn't stem from differences between tonal and nontonal languages. This is because our findings align with research (Faber & Fiveash, 2014) on a nontonal language as well. If language type were the key factor, all studies involving nontonal languages would consistently show no between-group differences, which is not the case.

p.20, l.412-414 “Thus, our findings suggest that instrumental music training may primarily influence the implicit, rather than explicit, processing of emotional prosody in tonal languages.” Comment 6: Why were these results obtained and what processes in people can be attributed to these results? More adequate discussions here should be useful to deepen author(s)’ findings.

**Response:** In this revised version, we have elucidated the underlying reasons for these results and the cognitive processes that can be attributed to them. We posit that statistical learning enables both music and non-music subjects to acquire implicit knowledge about the statistical regularities between acoustic cues and musical emotions through mere passive music exposure. Grounded in this implicit knowledge, all subjects exhibit a heightened attentiveness to those cues in emotional prosody and demonstrate the ability to make intensity assessments. However, nonmusic subjects do not attain the same level of attunement to the cues as their musically trained counterparts. This disparity stems from the fact that music subjects, through their focused and intensive training, develop an enhanced sensitivity to acoustic cues, rendering them particularly adept at detecting such regularities. Considering the implicit nature of statistical learning, its impact is more evident in implicit tasks rather than explicit tasks. (pp. 26-27, ll. 491-507)

p.20, l.417-418 “Future studies should employ true experimental designs to address this issue.” Comment 7: What is “true” experimental design? And how can it be achieved?

**Response:** The term "true experimental design" was intended to refer to a "randomized experimental design," which involves randomly assigning musically untrained individuals to treatment and control groups to establish causal relationships. We have replaced "true" with "randomized" to improve clarity and understanding.

p.20, l.420-421 “Third, this investigation utilized only one tonal language, raising questions about the generalizability of the findings to other tonal languages.” Comment 8: If there was a table, which could list the previous studies, it would be easier for the reader to understand the novelty of this study. For example, for each previous study, the language(s) covered, the type of musical experience and the age of the participants.

**Response:** In accordance with your suggestion, we have included supplemental S1 Table, which details the sample size, age, musical training (type and years), and language experience of the musical subjects participating in the research directly relevant to this study. (p. 4, ll. 71-74)

p.20, l.425-329 Comment 9: This last part of discussion can be separated Conclusion section. A succinct but sufficient summary of importance of this research, the development of future research (e.g. contrasts with Chinese and languages close to it are probably yet to be done), and the applications can provide stronger impressions to potential readers. In particular, how this research can be applied in real-life situations, social issues or human evolution?

**Response:** Incorporating your valuable feedback, we have emphasized the significance of this study, charted a course for future research, and delineated potential applications to enhance the manuscript's appeal to potential readers. (p. 28, ll. 518-530)
